# Transition of phase response properties and singularity in the circadian limit cycle of cultured cells

**DOI:** 10.1371/journal.pone.0181223

**Published:** 2017-07-17

**Authors:** Satoshi Koinuma, Hiroshi Kori, Isao T. Tokuda, Kazuhiro Yagita, Yasufumi Shigeyoshi

**Affiliations:** 1 Department of Anatomy and Neurobiology, Kindai University Faculty of Medicine, Osakasayama, Osaka, Japan; 2 Department of Information Sciences, Ochanomizu University, Bunkyo-ku, Tokyo, Japan; 3 Department of Mechanical Engineering, Ritsumeikan University, Kusatsu, Shiga, Japan; 4 Department of Neuroscience and Cell Biology, Kyoto Prefectural University of Medicine, Kamigyo-ku, Kyoto, Japan; University of Texas Southwestern Medical Center, UNITED STATES

## Abstract

The circadian system has been regarded as a limit cycle oscillator constructed by the integrated interaction of clock genes and proteins. Here, we investigated a mammalian circadian oscillation geometrically before and after a perturbation. We detected the singular point and transition from a type 1 to type 0 phase response curve (PRC) and determined the embedding dimension to show how many variables are needed to describe the limit cycle oscillation and relaxation process after a perturbation. As a perturbation, forskolin (FK) was administered to Rat-1 cells expressing the *Per2*::*luc* gene. By broadly and finely changing the phase and strength of the perturbation, we detected the transition of the PRC from type 1 to type 0 and a possible singular transition point, the property of which agreed quite well with our numerical simulation of the noisy Goodwin model, a simple yet canonical model for the transcription-translation feedback loop of the core clock genes. Furthermore, we estimated the embedding dimension of the limit cycle before and after the perturbation. The trajectory of the limit cycle was embedded in two dimensions but with the perturbation of the state point moved out of the trajectory, the relaxation process was generally embedded in higher dimensions. The average number of embedding dimensions at each dose of FK increased as the FK dose increased but most of the relaxation process was generally embedded within four dimensions. These findings support the existence of a circadian limit cycle oscillator in mammalian cells and suggest that a small number of variables determine the relaxation process after a perturbation.

## Introduction

The mammalian cells of the biological clock oscillate with a stable circadian period. The oscillation of these circadian clock cells is regulated by a transcription-translation negative feedback loop [1, 2]. At the molecular level, E-box binding transcription factors such as *Per (Per1/2)* and *Cry (Cry1/2)* repress their own expression through directly inhibiting BMAL1 and CLOCK, which positively regulate *Per* and *Cry*. PER and CRY are subsequently degraded by proteasomes [3–5]. The negative regulations generate a stable periodic oscillation of the clock genes. The system underlying this oscillation can be described as a limit-cycle oscillator, which forms an isolated closed orbit in a phase space [6–8].

**Fig 1:**
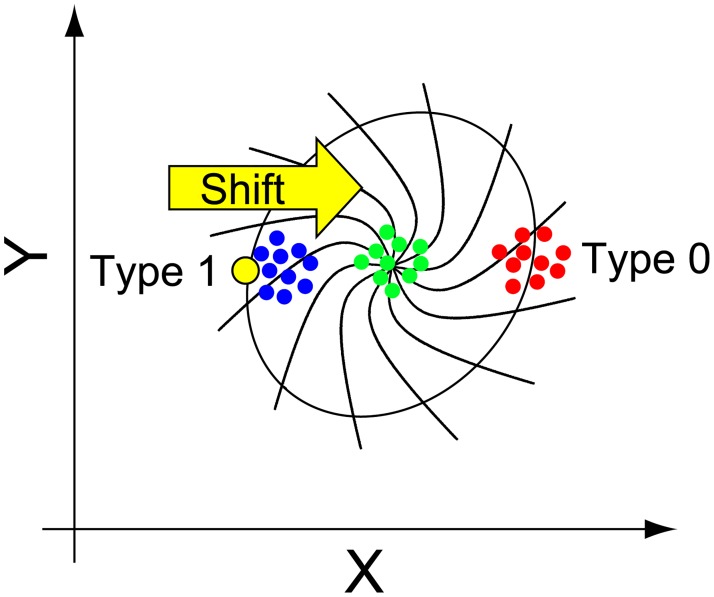
Conceptual diagram of the limit cycle oscillator. Suppose a set of variables X and Y is in circadian oscillation, where X is instantaneously upregulated by a certain level of perturbation, and the phase of Y is affected concomitantly by the amount of X. In the two-dimensional system, a state point (X, Y), colored in yellow, circulates periodically in the phase plane. A unique point in the center of the trajectory is the assumed singular point. Lines radiating from the singular point are isochrones, which correspond to equal phases. When a perturbation (yellow arrow) is applied to the oscillator, the oscillator deviates from the trajectory and reaches a different isochrone (weak perturbation, blue dots; medium perturbation, green dots; strong perturbation, red dots).

Limit cycle oscillators have several properties [6, 9]. First, the closed orbit is asymptotically stable (Fig 1). The oscillator deviates from the closed orbit after a perturbation is applied to the system, but subsequently returns to the closed orbit. In this case, the oscillator recovers its amplitude and period, but generally, the phase of oscillation is shifted compared with that without perturbation. From this fact, the notion of phase can be extended to the entire phase space as described below. The set of initial points in the phase space converging to an identical point on the limit cycle is regarded to have an identical phase, and the set is referred to as an isochrone (Fig 1). Second, there are sets of points in which the phase cannot be defined, which are referred to as phaseless sets or singular points. In a two-dimensional system, there is typically a singular point inside a limit cycle to which all isochrones converge (Fig 1). Singular points are practically important because once the state is put around one of these points by a perturbation, the information about phase will be lost. Singular points also play a critical role in determining qualitatively different types of phase response, referred to as type 0 and type 1 phase response curves (PRCs) (detailed later).

Various studies in relation to the perturbation magnitude and the resulting phase have been explored. Winfree schematically depicted in the three-dimensional lattice that the transition from type 0 to type 1 under increasing magnitude of stimuli occurs discontinuously by using data from insects and plants [10]. In the experiments, as the perturbation duration lengthened or the magnitude strengthened, type 1 to type 0 transitions were observed. Furthermore, in *Gonyaulax*, no fixation of the phase was observed even in the type 0 PRC. With strengthening of the perturbation magnitude, a breakpoint (discontinuous region observed in type 0 PRC) moves to an earlier phase (leftward) in a dose-dependent manner [11]. Thus, not only the timing of the perturbation but also the magnitude determines the final phase after perturbation.

In the mammalian biological clock, most of the phase response at an organism level is exhibited as type 1 PRC, with some exception, such as in humans, where some individuals show type 0 PRC after repeated bright light exposure [12] and in hamsters, homozygous *tau* mutant exhibited type 0 PRC after single 1-hr light pulses [13]. In contrast to the organism level, mammalian tissues or cultured cells showed type 0 resetting. It was suggested that the magnitude of the phase shift correlates with the amplitude of the residual circadian oscillator [14, 15]. This is theoretically considered as the large diameter of the limit cycle, i.e., a large amplitude of the circadian oscillator, is less likely to be affected by a constant perturbation than a smaller one at the single cell level.

Phase responses of cultured cells in combination with various perturbation agents have been studied intensively. PRCs of mammalian cells are largely classified into two groups based on up- or down-regulation of *per1* expression, i.e., a light-pulse or a dark-pulse PRC. A wide variety of stimuli such as forskolin (FK) [16], serum [17], and dexamethsone [18] exerted phase resetting as the light-pulse PRC type, whereas a few such as glucose [19] and prostaglandin J_2_ [20] were reported to show dark-pulse PRC. Another type of PRC was also demonstrated whereby ionizing radiation exclusively phase-advanced a circadian rhythm in Rat-1 fibroblast cells [21].

Employing clock gene-driven bioluminescence reporter assays, it is possible to record the cellular circadian rhythm in a high-throughput manner. Combined with a photo-perturbation system, Ukai et al. demonstrated that desynchronization of individual cellular clocks underlies singularity behavior in cultured cells [22]. Although the idea of the singular point is widely accepted, the precise behavior of a state point on the limit cycle in response to a perturbation signal around the singular point is unclear in mammalian circadian cells.

*Per1* and *Per2* are deemed as state variables in the circadian limit cycle oscillator [8, 23]. Many reports have suggested that induction of *Per1* or *Per2* genes by the perturbation causes a phase shift [24–28]. Thus, it has been reported that in the suprachiasmatic nucleus, the mammalian central circadian oscillator, induction of *Per1* and *Per2* through the N-methyl-D-aspartate receptor and pituitary adenylate cyclase-activating peptide receptor leads to a phase shift [29, 30]. Up-regulation of *Per1* gene is dependent on cyclic adenosine monophosphate (cAMP) signaling via the cAMP response element site in the flanking region of *Per1* [31], while *Per2* is dependent on Gq protein-coupled signaling [32]. This machinery is conserved within other peripheral tissues and various cell lines [17, 18, 33]. We have previously comprehensively investigated the enhancer elements and transcription factors involved in the circadian feedback loop [34]. Presently, more than 20 genes have been reported to be involved in this loop. Considering the proteins and modification forms involved, it is also assumed to form a set of state variables, which could result in a large number of possible state variables. However, no study has demonstrated how many of these are independent variables.

In the present study, we characterized a mammalian circadian limit cycle geometrically, by detecting the singular point and transition from type 1 to type 0 PRC and determined the embedding dimension to show how many variables are needed to describe the limit cycle oscillation and relaxation process after a perturbation.

## Results

### Computational simulation of the limit cycle oscillator during perturbation and relaxation processes

**Fig 2:**
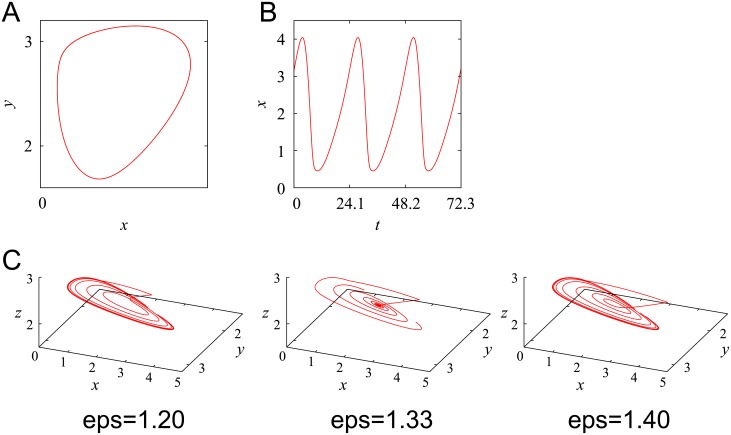
Computational simulation of the limit cycle and relaxation processes after perturbation without noise. (A) Limit cycle in (*X*, *Y*) plane and (B) time series of X(*t*) for the unperturbed system (Eq (1)). (C) Trajectories for 0 ≤ *t* ≤ 164.3 in (*X*, *Y*, *Z*) space, in which perturbation was given during 13.3 < *t* < 14.3. Parameter values were the same as those employed in Locke et al. [36]: *v*_*1*_  =  6.8355, *n*  =  5.6645, *K*_*1*_  =  2.7266, *v*_*2*_  =  8.4297, *K*_*2*_  =  0.2910, *k*_*3*_  =  0.1177, *v*_*4*_  =  1.0841, *K*_*4*_  =  8.1343, *k*_*5*_  =  0.3352, *v*_*6*_  =  4.6645, *K*_*6*_  =  9.9849, *k*_*7*_  =  0.2282, *v*_*8*_  =  3.5216, *K*_*8*_  =  7.4519, *v*_*c*_  =  6.7924, *K*_*c*_  =  4.8283, *K*  =  1, *L* = 0.

We employed a mathematical model proposed previously [35], given by Eq (1), where parameter values were inferred from real data [36]. We introduced noise terms for all variables to the original model, where *μ* is noise intensity and *ξ*_*i*_ = (*i* = *X*, *Y*, *Z*, *V*) is Gaussian white noise with zero mean and unit variance. In the absence of noise (i.e., *μ* = 0), for general initial conditions, the system falls into a periodic orbit, i.e., the limit cycle (Fig 2A and 2B). The period was *τ* ≃ 24.1 [h]. The phase (i.e., circadian time, CT) of the state on the limit cycle was defined as follows: if the state was the same as that at *t* in Fig 2B (0 ≤ *t* ≤ *τ*), we regarded the phase of the state as CT = *t*. To mimic the effect of FK, perturbation to the system was given by replacing the first term of Eq (1a) with *εv*_*1*_ for the duration *t*_p_ *≤* *t <t*_p_ + 1, where *t*_p_ (0 ≤ *t*_p_ ≤ *τ*) is the timing of the perturbation. Because CT and *t* are the same in the unperturbed system, the perturbation was provided when CT = *t*_p_. Fig 2C displays the trajectories (0 ≤ *t* ≤ 164.3) for three different perturbation strengths *ε*, where the perturbation was given at *t*_p_ = 13.3. For *ε* = 1.33, the system closely approached the unstable fixed point (i.e., the singular point) and spent a long time near it.

$$\frac{dX}{dt}=v_{1}\frac{K_{1}^{n}}{K_{1}^{n}+Z^{n}}-v_{2}\frac{X}{K_{2}+X}+v_{c}\frac{KV}{K_{c}+KV}+L+\mu \xi_{x}$$ (1a)

$$\frac{dY}{dt}=k_{3}X-v_{4}\frac{Y}{K_{4}+Y}+\mu \xi_{Y}$$ (1b)

$$\frac{dZ}{dt}=k_{5}Y-v_{6}\frac{Z}{K_{6}+Z}+\mu \xi_{Z}$$ (1c)

$$\frac{dV}{dt}=k_{7}X-v_{8}\frac{V}{K_{8}+V}+\mu \xi_{V}$$ (1d)

**Fig 3:**
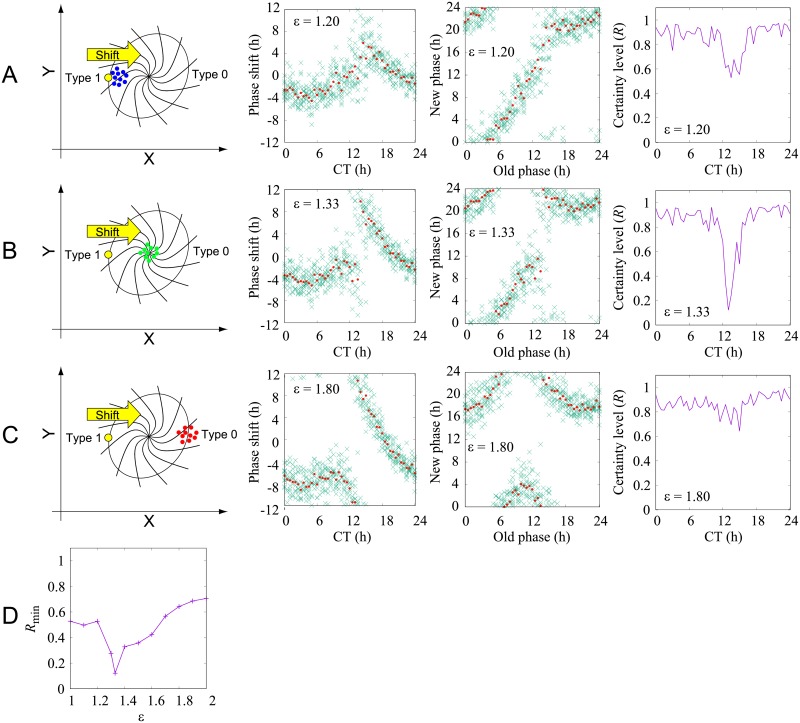
Computational simulation with noise and conceptual scheme of a state point behavior after several intensities of perturbation. (A–C) Schematic drawings of a limit cycle attractor and a perturbation delivered to the system are shown in the left column. An oscillator (yellow) circulates in the phase plane (X, Y) as shown in Fig 1. A unique point in the center of the trajectory was the assumed as a singular point where all isochrones converge. When perturbations of varying intensity (yellow arrow) were applied to the oscillator, the oscillator deviated from the trajectory by various distances. (A) In the weak intensity case (blue dots), the oscillator moved in the vicinity of the trajectory where the phase difference was small (type 1 phase resetting). (B) In the moderate case (green dots), the oscillator reached close to the singular point. (C) In the strong case (red dots), the oscillator moved to the opposite area of the trajectory, giving the large phase shift (type 0 phase resetting). The middle column shows the phase response φ_*j*_ (*j*  =  1, …30) for each perturbation timing CT (crosses) and mean phase Φ (CT) (filled circles). The right column shows the certainty level *R* (CT). (D) Minimum of *R* for each *ε* value.

Next, we measured the PRC using the model. We choose the noise intensity *μ* = 0.05. With this choice, the standard deviation of a cycle-to-cycle period was about 1.7 [h], being close to the real variability of a free-running period [37]. The initial condition was the same as that in Fig 2B. Perturbation was given at *t*   = *t*_p_ (i.e., when CT = *t*_p_). To measure the phase correctly, we turned off noise for *t* > 48 and measured the resulting phase shift at *t* = 96. We performed *M* = 10 different realizations for each *t*_p_ (Fig 3A–3C). The obtained PRCs for *ε* = 1.20 and *ε* =  1.80 are qualitatively different, usually classified as type 1 PRC (winding number = 0) and type 0 PRC (winding number = -1), respectively [38, 39]. Typically, the former and the latter are obtained for weak and strong perturbations, respectively. The transition between them was observed at around *ε*  = 1.33. As depicted in Fig 3B, at the transition point, perturbation given at a certain CT leads to a singular point. These data were analyzed statistically using Eq (2), where *R* (0 ≤ *R* ≤ 1) and Φ(−*τ*/2 < Φ ≤ *τ*/2) corresponded to the Kuramoto order parameter and the mean of the phase shifts φ_*j*_ (*j* = 1, …, *M*), respectively, at each perturbation timing CT (Fig 3A–3C) [40]. We obtained *R* = 1 when the obtained phase shifts φ_*j*_ (*j* = 1, …, *M*) were the same and *R* = 0 when φ_*j*_ were distributed uniformly. The Kuramoto order parameter is usually used for quantification of the level of phase synchrony of a population of oscillators. In this paper, we used this quantity to measure the level of certainty of the phase response. At the transition point *ε* ≃ 1.33 and CT ≃ 13, *R* was considerably smaller, implying that the phase response was highly variable. In Fig 3D, the minimum value of *R* for each perturbation strength *ε* was displayed.

$$R\text{exp}\left[\text{i}\frac{2\pi \Phi }{\tau }\right]=\frac{1}{M}\Sigma_{j=1}^{M}\text{exp}\left[\text{i}\frac{2\pi \varphi_{j}}{\tau }\right]$$ (2)

### *In vitro* experiments of phase response against various intensities of perturbation

**Fig 4:**
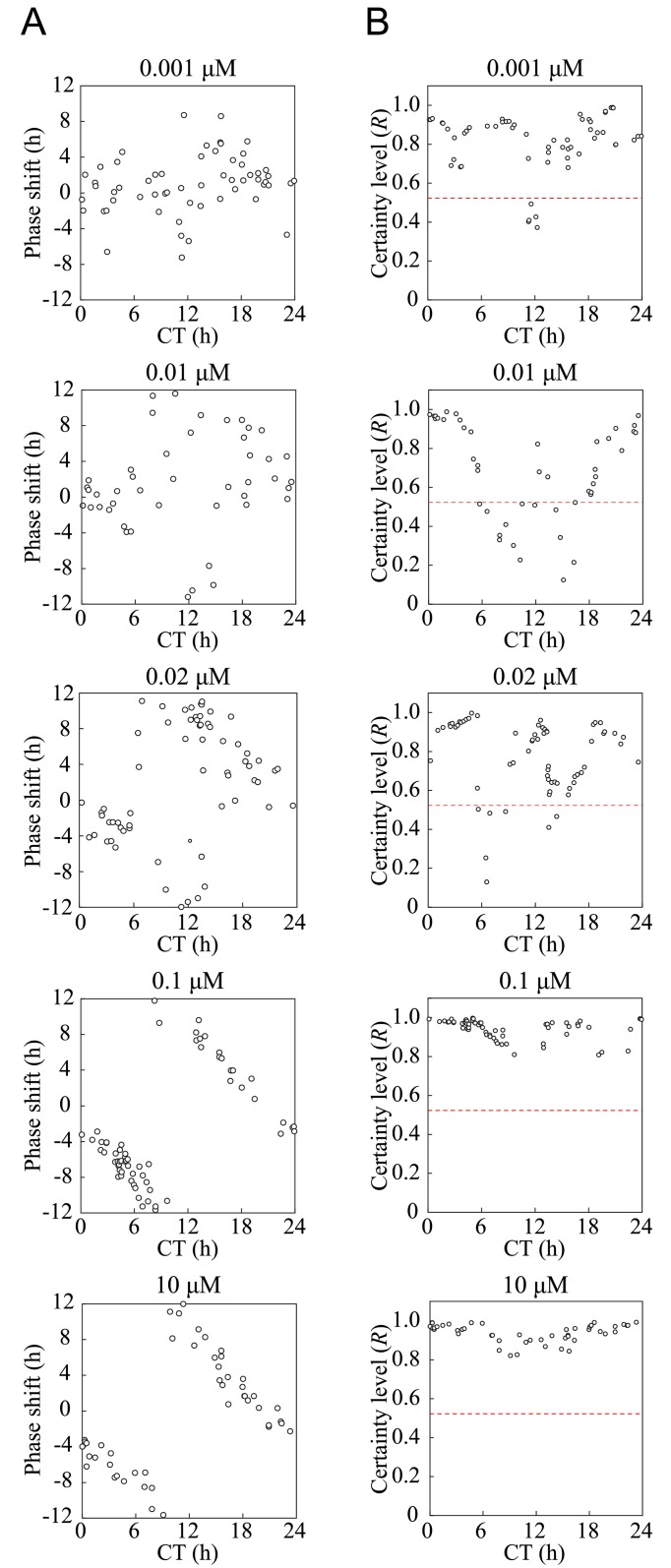
Phase response curves and phase diversity after weak to strong perturbation in Rat-1 m*Per2*::*luc* cells. (A) Phase response curves (PRCs) of Rat-1 m*Per2*::*luc* cells treated with five different concentrations (0.001 μM, 0.01 μM, 0.02 μM, 0.1 μM, and 10 μM) of forskolin (FK). As the concentration of FK increased, traces of PRCs changed from type 1-like to type 0-like resetting curves. At the medial concentration (0.01, 0.02 μM), various magnitudes of phase shift were observed. (B) The certainty level *R* was calculated from five nearby data points of the PRC data. Above the critical line (*R*_*c*_ = 0.522, red dashed line), the perturbed phases are significantly correlated with the nearby phases (V-test, *p* = 0.05).

In order to investigate the topological characteristics of the limit cycle in the mammalian circadian rhythm, we first examined whether particular aspects of the limit cycle could be observed in experiments using cultured cells (Rat-1 m*Per2*::*luc*). We carried out phase-shifting experiments by administering five different concentrations (0.001 μM to 10 μM) of FK to the cells at various phases. As predicted by the mathematical model (Fig 3A–3C), weak perturbation (0.001 μM) showed a relatively small shift in phase between CT9 and CT12. In contrast, intense stimulus (0.1–10 μM) exerted a large shift between CT9 and CT12. Because the PRC of 0.1 μM and 10 μM FK showed discontinuous PRC, it was probable that type 0 phase resetting occurred for these FK concentrations. Notably, we observed a large variability of phase-shifts between CT12 and CT18 for 0.01 μM FK (Fig 4A). This was confirmed by the phase variance analysis (Fig 4B and S1 Fig), which indicated the lowest *R*-value at CT15, suggesting that the system was perturbed close to the singular point. In contrast, the phase shifts showed a small variability among nearby phases for 0.1 μM and 10 μM FK (Fig 4B, *R* ≈ 1.0 for all perturbation timings) and for 0.001 μM FK (Fig 4B, *R* > 0.7 for most perturbation timings). Above the critical line (red dashed line of Fig 4B), the perturbed phases are significantly correlated with the nearby phases (V-test, *p* = 0.05) [41]. Below the critical line, on the other hand, the phases are decorrelated, possibly due to the singularity. These data suggested that transition between type 0 and type 1 PRCs emerged at around 0.01 μM and 0.02 μM.

**Fig 5:**
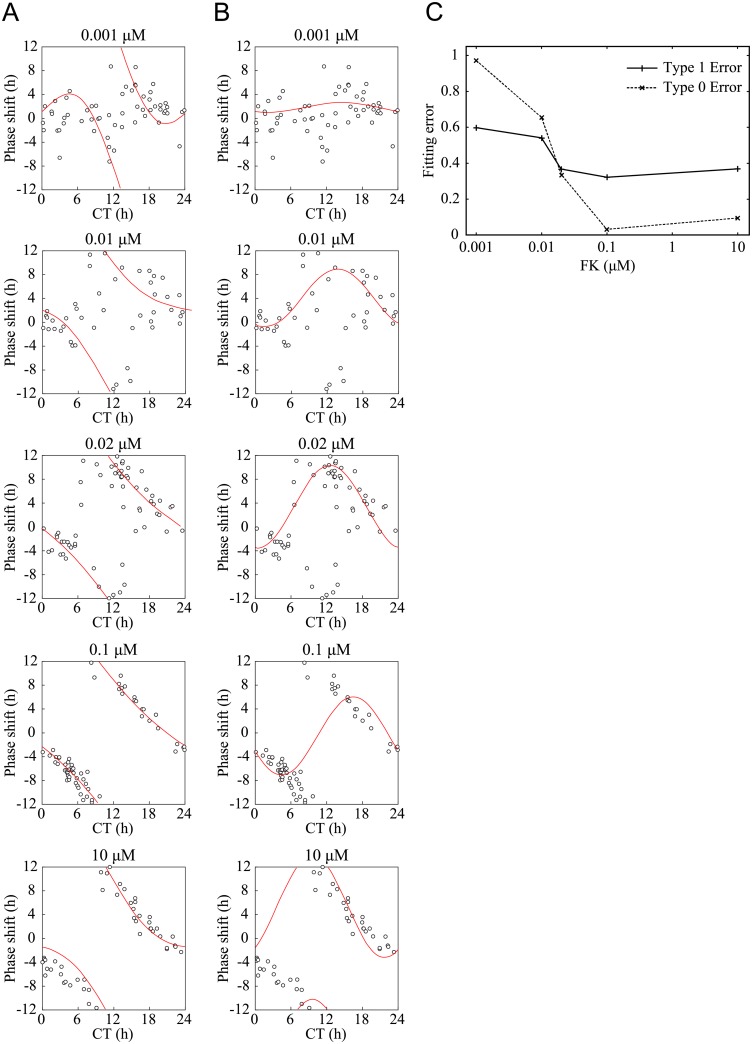
Fittings to phase response curves revealing the transition point from type 1 to type 0 resetting. (A–B) The experimental phase response curves (PRCs) data (Fig 4) was fitted to a type 0 fitting curve (A) and to a type 1 fitting curve (B). (C) The graph shows the fitting errors to the PRCs of Rat-1 m*Per2*::*luc* cells. The crossing point of the two lines of type 1 and type 0 fitting errors represents the transition point of the two types of PRC. The transition point was between 0.01 μM and 0.02 μM. The intensity of the perturbation (concentration of forskolin) is shown on the x-axis (logarithmic) and the fitting error is plotted on the y-axis.

To further corroborate the transition of PRC, the PRC were fitted to type 0 and type 1 functions (S1 Methods). Fig 5 shows the results of the PRC fittings applied to the experimental data. As the FK concentration increased, the fitting error E_*nrms*_ decreased for both type 0 and type 1. This was because stronger stimuli reduced the noise components discernible in the experimental PRCs. Such noise produced fitting errors especially when the stimuli were weak. For weak stimuli, the error for type 1 was smaller than that for type 0. At FK of 0.02 μM, this relationship was reversed and the type 0 fitting became superior. This agreed quite well with the visual inspection of the fitted curves in Fig 5, where stronger stimuli produce a clear breakpoint in the PRCs. Consequently, the experimental data with stronger stimuli were well approximated by type 0 PRCs. The present analysis, therefore, implied that the transition from type 0 to type 1 PRC occurs around an FK value of 0.02 μM.

### Minimum embedding dimension of experimental time series data

**Fig 6:**
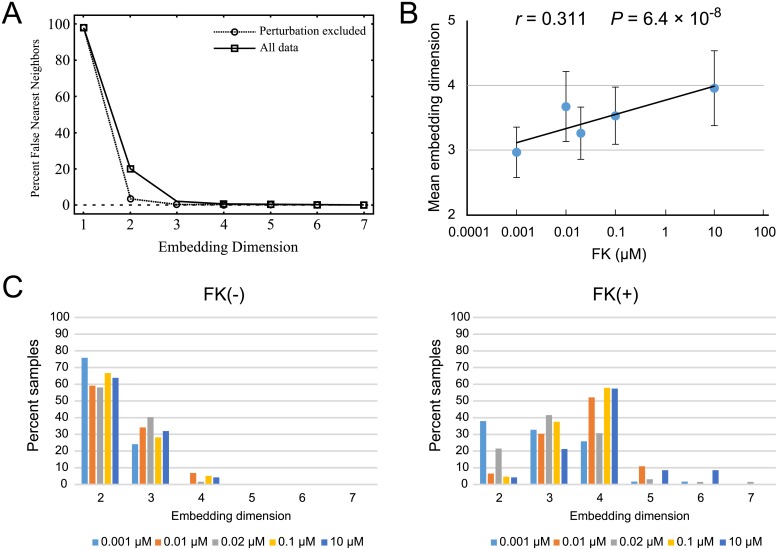
False nearest neighbor analysis applied to the time series datasets of the phase shift experiments. (A) A representative diagram of the false nearest neighbor (FNN) analysis after 10 μM forskolin (FK) administration. The embedding dimension is shown on the x-axis and the “false” possibility is indicated on the y-axis. In this case, zero percent false is shown at three dimensions in the analysis without perturbation, thus, its dimension is embedded within two dimensions. For the analysis including both perturbation and relaxation processes, zero percent is shown at four dimensions, thus, it requires three dimensions to unfold all the delayed time series data. (B) The means of the embedding dimensions were calculated for the time series datasets of each FK concentration. As the intensity of perturbation increased, the embedding dimension rose. (C) Percentages of the embedding dimensions in the case of excluding the perturbation signals (FK (−)) and in the case of using all data (FK (+)). Most populations of the embedding dimension in FK (−) were embedded below two dimensions. In contrast, for FK (+) samples, the majority were in three to four dimensions. Colors of bars indicate FK concentration (light blue, 0.001 μM: orange, 0.01 μM: gray, 0.02 μM: yellow, 0.1 μM: blue, 10 μM).

Throughout the phase-shifting experiments, we collected a large amount of bioluminescence signals including the perturbation and relaxation processes for a wide variety of phases and perturbation strengths. We utilized these data to gain a topological insight into the coordinate space where the circadian limit cycle oscillator exists. In order to investigate how many state variables (dimensions) constitute the circadian limit cycle trajectory, we analyzed the minimum embedding dimensions of the bioluminescence signals. Fig 6 shows the results of a false nearest neighbor (FNN) method (S2 Methods) applied to the luminescence signals from Rat-1 m*Per2*::*luc* cells treated with FK at various phases or without FK. The time lag was selected as *τ* = 6 h, which gave the first zero-crossing point of the auto-correlation function of the signal. The threshold value was set as *R*_tol_ = 10 and the reconstruction dimension was varied from *d* = 1 to *d* = 7. In this analysis, only the first nearest neighbor was considered, i.e., *r* = 1.

Concerning our setting of *τ* = 6 h, which corresponds to one fourth of the 24 h period, it has been cautioned that this sort of time lag selection may produce a spurious correlation in the embedding space, potentially altering the results [42]. In our data from Rat-1 m*Per2*::*luc* cell bioluminescence, the periods were 23.57 ± 0.05 h and 23.53 ± 0.07 h (mean ± SEM, *n* = 280) before and after the FK administration, respectively. Since the periods only deviated slightly from 24 h, the problem of the correlated embedding may not be too serious. We have furthermore examined the cases of *τ* = 5 h and *τ* = 7 h and confirmed that the results remained essentially the same.

As the reconstruction dimension increased from *d* = 1, we saw that the percentage of false nearest neighbors decreased and reached almost zero for reconstruction dimensions higher than *d* = 4 for the signals before FK was administered (Fig 6A–6C). However, the dimension rose when FNN analysis was applied to signals that included the perturbation and relaxation process (Fig 6C). The mean reconstruction dimension in FK(−) samples was 2.38 ± 0.03 and that in FK(+) was 3.45 ± 0.05 (mean ± SEM, *n* = 280, *P* = 2.07 × 10^−54^, paired Student’s *t*-test). Furthermore, there was a significant correlation between the perturbation intensity and the reconstruction dimension (*r* = 0.311, *P* = 6.4 × 10^−8^, Fig 6B).

## Discussion

In the present study, we demonstrated that the circadian rhythm generated in Rat-1 fibroblasts has a singular point. When a limit cycle oscillator receives a perturbation that shifts the state variable close to the singular point, the phase becomes very sensitive to a slight difference in perturbation and takes a variety of phases. This randomized property of the perturbed phases was indicated by the low value of *R* in our present study. One more finding that supported the existence of the singularity was the transition of PRC from type 1 to type 0 with increasing stimulus strength. These findings suggested that a limit cycle oscillator generates the mammalian circadian rhythm in single cells and that FK administration followed by clock genes induction was able to shift the state point very close to the singular point of the limit cycle.

In contrast to the relatively small fluctuations observed in the phase shift induced by a strong stimulation that produced a type 0 PRC, a weak perturbation that produced a type 1 PRC showed large fluctuations compared with the amount of the phase shift. It is possible that the experimental manipulation yielded random noise that resulted in rather large fluctuations after the weak perturbation. With a strong perturbation, the amount of the shift was large, so the noise levels might have been almost negligible. However, with a small stimulation, the noise might have been large compared with the amount of the shift, so this would have affected the results. We interpreted the PRC of small stimulation as type 1 based on the functional fitting error in comparison to that of type 0. It should be noted, however, that the functional fitting provides a mere guideline on an abstract location of the transition point between type 0 and type 1 PRCs. In general, delicate procedure is needed to identify PRC when the noise level is relatively high [43]. Under such condition, the transition point becomes unclear, making the identification of the exact point very difficult. It is an important future task to classify the type of PRCs more reliably in the presence of noise.

In the present study, we employed a single-cell model to simulate a phase response of the limit cycle to various strengths of perturbation. Although the simulation was calculated on single-cell basis, the experiment was done at the population-level to obtain the detectable bioluminescence intensity. Among the traces indicated in S2 and S3 Figs, some of the amplitudes were remarkably weakened after adding low concentration FK. Under weak and near-singularity perturbation strengths there was likely a spread of phases within the population possibly due to variability in the concentration of FK in the culture dish. However, the simulated PRC showed good agreement with the experimental PRC. We considered that the magnitude of phase shift at the population level could be represented as that of a single cell for the following reasons. First, cultured fibroblasts do not show significant cell-to-cell coupling [44]. Second, the amplitude of the circadian rhythm of individual fibroblasts, including Rat-1 cells, is not damped even after several weeks [37, 45]. Thus, the relative strength of perturbation to the limit cycle does not change over the course of the experiment. Third, because we gave a strong stimulus that exerted a type 0 phase, resetting the cells, they started to oscillate from an equivalent phase. Taking into account that FK was administered before the seventh cycle after the initial resetting, the majority, if not all, of the cells were still synchronized at that point. Taken together, we presumed a phase analysis in which the population-level Rat-1 cells represent a limit cycle behavior of individual cells. To more accurately know the degree of desynchronization, it will be necessary to measure the bioluminescence rhythm of the whole cell population with a CCD camera using cells that produce strong bioluminescence.

Before stimulation by FK, the circadian oscillation of the cell was shown to be embedded in a two-dimensional space, which suggested that the circadian limit cycle attractor indeed has two degrees of freedom. After FK application, the relaxation process gave rise to higher dimensional dynamics, which were embedded in three or four dimensions. This suggested that, although the state points jumped out of the two-dimensional plane, in which the limit cycle attractor of the circadian rhythm was confined, only an few additional variables played roles in its relaxation processes. Mammalian cells shift their circadian rhythm by shifting state variables nonparametrically, such as acute induction of either *Per1* or *Per2* genes, or both [24, 28, 46]. In the present observation, the state variable jumped out of the attractors and returned back to the stable attractor after several cycles. FK induced *Per1* transcription but not *Per2* [32, 47], so it is likely that *Per1* induction by itself makes the state point jump out to three or more dimensions. In a few cases, after the perturbation by FK, the embedding dimension increased to five or more. This suggested that by the FK stimulation the state point jumped out of the circadian limit cycle attractor and a greater number of state variables such as clock genes and proteins contributed to the relaxation process and to the appearance of higher dimensions.

Conversely, considering the number of clock genes reported so far, the embedding dimension was rather small even after FK stimulation, being at most seven dimensions. In the present study, in order to describe the dynamics numerically, we used differential equations based on the model constructed by Goodwin [48, 49]. This model has been applied to various problems in chronobiology because of its simplicity with a few dynamical variables. The embedding dimension study suggested that the complexity of the dynamics of the circadian rhythm with or without perturbation can be reproduced by models with a few dynamic variables such as the Goodwin model.

## Materials and methods

### Cell culture

Rat-1 m*Per2*::*Luc* fibroblast cells, which contain a 4.1 kb upstream region of the m*Per2* gene tethered to a firefly luciferase gene [50], were plated in 35 mm dishes with Dulbecco's modified Eagle's medium (DMEM; Sigma, St. Louis, MO) supplemented with 10% fetal bovine serum (FBS; ICN, Cleveland, OH) at 37°C in a 5% CO_2_ atmosphere. Once they reached confluence, cells were treated with dexamethasone (100 nM; Wako, Osaka, Japan) for 1 hour to synchronize the oscillation. The medium was replaced with DMEM containing 25 mM HEPES without phenol red (Sigma, St. Louis, MO) to wash out dexamethasone. Luciferin potassium salt (200 μM; Wako, Osaka, Japan) was added to the medium and the dish was sealed with a thermoplastic, self-sealing film (Parafilm M; Bemins, Oshkosh, WI) prior to the measurement of bioluminescence. Luminescence was monitored under photomultiplier tubes (PMT, ATTO, Tokyo, Japan, and Hamamatsu Photonics, Shizuoka, Japan) [50]. FK (10 μM; Wako, Osaka, Japan) was added to the medium at various phases between the fourth and seventh oscillations from the start of monitoring.

### Phase response curves

Traces of luminescence were detrended by subtracting a 3-hour running average to 24-hour running average [51]. An exponentially damped sine curve $x=x_{0}+Ae^{\left(-t/t_{0}\right)}\text{ sin}\pi (\left(t-t_{c}\right)/w)$ was fitted to each detrended trace both 72 to 24 hours before the FK treatment and 24 to 72 hours after treatment. The functional fitting was done using Origin 8.1J software (OriginLab, MA, USA) [52]. We confirmed goodness of fit by the adjusted r-square value to > 0.9. There were two instantaneous phases calculated by the fitting of pre-FK and post-FK treatment. The magnitude of the phase shift was calculated by subtraction of these two phases. We defined the peak of the bioluminescence rhythm as CT6.

## Supporting information

S1 FigCircadian time of the most diverse phase response, analyzed by cosine fitting to the level of certainty (*R*).(A) A dataset of the certainty levels (calculated from −1.5 h to +1.5 h of each PRC point) was fitted by a cosine curve $\left(\text{y}=y_{0}-A\text{cos}\left\{\frac{\pi \left(x-x_{c}\right)}{12}\right\}\right)$. The troughs of the fitted curves indicated the levels of certainty (*R*) and the phase of the most diverse phase response (x_c_) to the perturbation. (B) The mean levels of certainty (*R*) at each forskolin (FK) concentration were plotted. The lowest mean *R* was 0.678 in 0.01 μM FK.(PDF)

S2 Fig Examples of raw and detrended traces of *Per2*::*luc* bioluminescence and damped sinusoidal curve fittings (0.001 μM and 0.01 μM forskolin). Raw and detrended traces of *Per2*::*luc* bioluminescence at various forskolin (FK) concentrations and circadian times (CT) within the shaded region of the phase response curve (PRC) are shown. A red bar represents the timing of FK administration. CT and the amount of phase shift are indicated above the traces. Damped sinusoidal curve fitting before and after FK administration are shown on the right.
(PDF)

S3 Fig Examples of raw and detrended traces of *Per2*::*luc* bioluminescence and damped sinusoidal curve fittings (0.02 μM forskolin). (PDF)

S4 Fig Examples of raw and detrended traces of *Per2*::*luc* bioluminescence and damped sinusoidal curve fittings (0.1 μM and 10 μM forskolin). (PDF)

S1 Dataset The detrended bioluminescence records from Rat-1 *Per2*::*luc* cells (0.001 μM, 0.01 μM, and 0.02 μM forskolin). (XLSX)

S2 Dataset The detrended bioluminescence records from Rat-1 *Per2*::*luc* cells (0. 1 μM and 10 μM). (XLSX)

S1 Methods Function fitting to phase response curves. (PDF)

S2 Methods Minimum embedding dimension of experimental time series data. (PDF)
