# Supplementary figures and images for: Transition of phase response properties and singularity in the circadian limit cycle of cultured cells
Source: PLoS One. 2017 Jul 17;12(7):e0181223. doi: 10.1371/journal.pone.0181223 (PMC5513448; doi:10.1371/journal.pone.0181223)

A

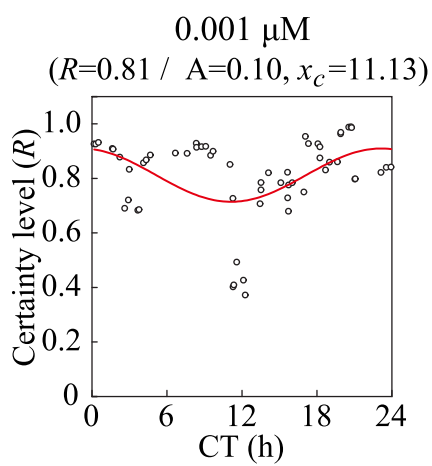

B

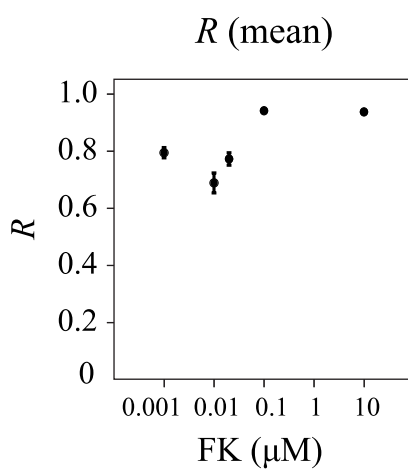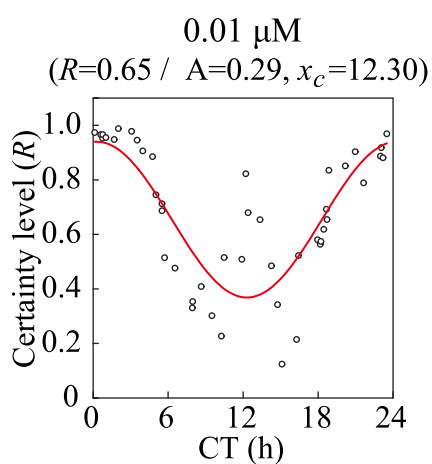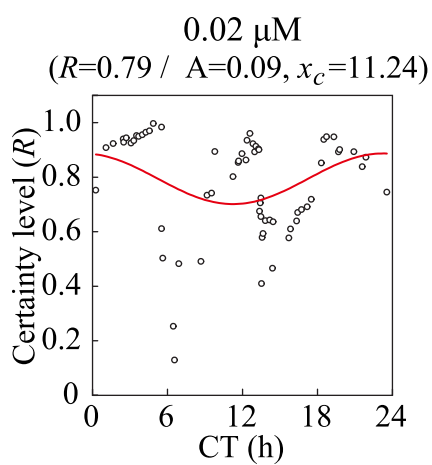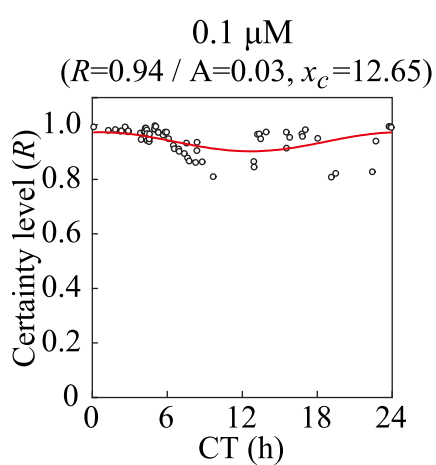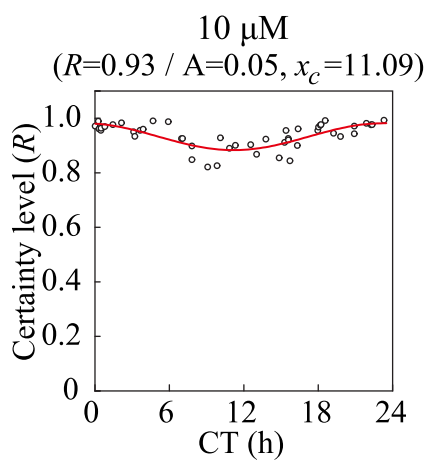

Supplement: S1 Fig — (A) A dataset of the certainty levels (calculated from −1.5 h to +1.5 h of each PRC point) was fitted by a cosine curve (y=y0−Acos{π(x−xc)12}). The troughs of the fitted curves indicated the levels of certainty (R) and the phase of the most diverse phase response (xc) to the perturbation. (B) The mean levels of certainty (R) at each forskolin (FK) concentration were plotted. The lowest mean R was 0.678 in 0.01 μM FK. (PDF) [file pone.0181223.s001.pdf]

FK (0.1  $\mu$ M)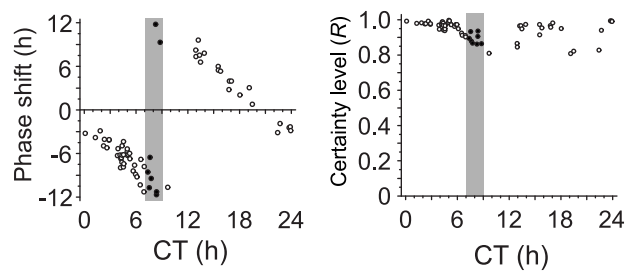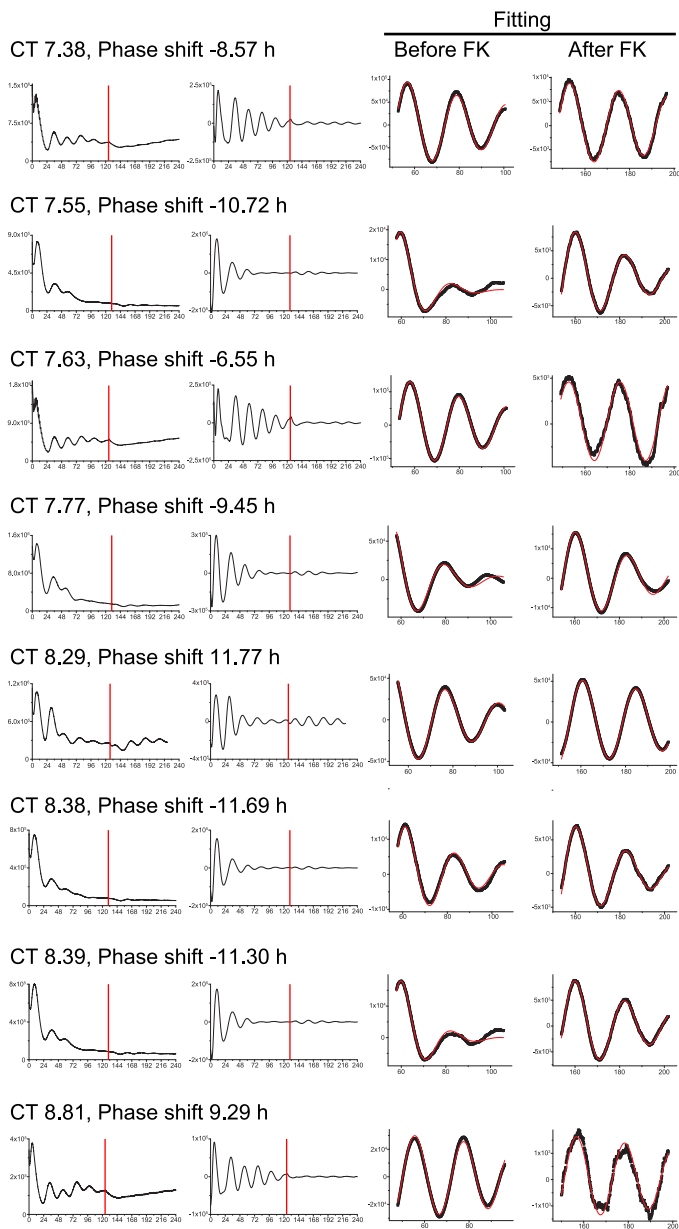FK (10  $\mu$ M)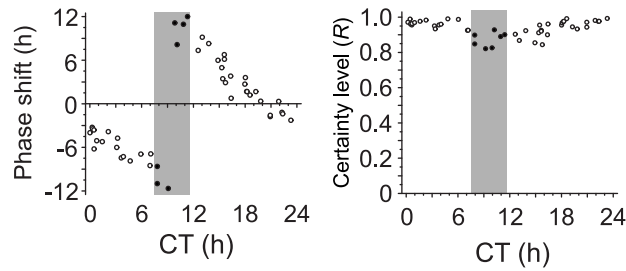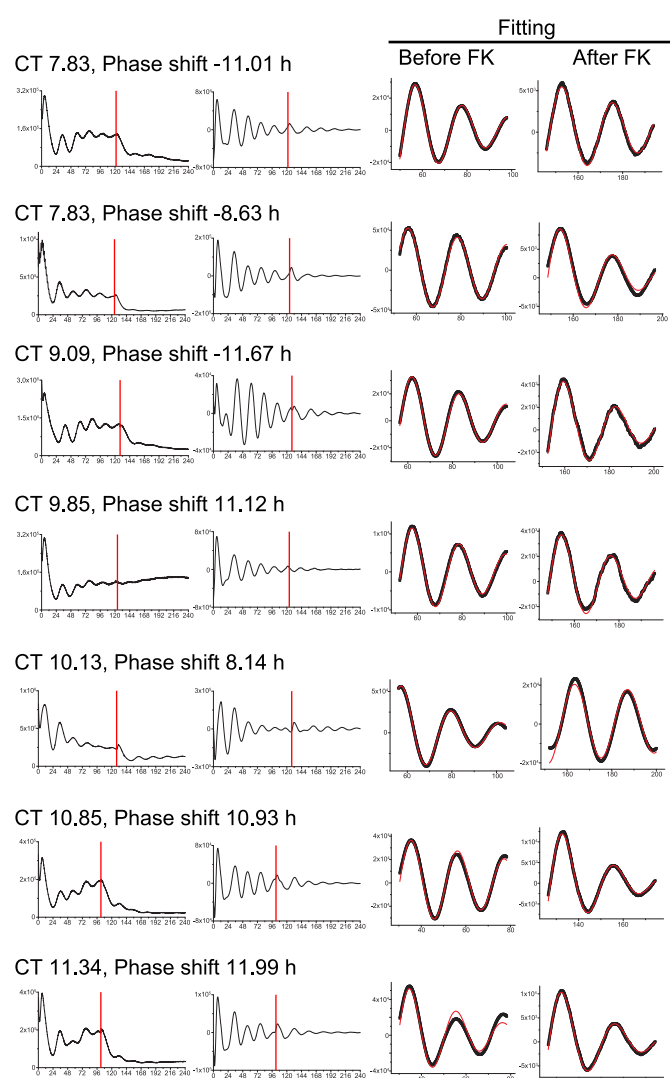

Supplement: S4 Fig — (PDF) [file pone.0181223.s004.pdf]
