# Supplementary material for: Transition of phase response properties and singularity in the circadian limit cycle of cultured cells
Source: PLoS One. 2017 Jul 17;12(7):e0181223. doi: 10.1371/journal.pone.0181223 (PMC5513448; doi:10.1371/journal.pone.0181223)
Supplement: S2 Fig — Raw and detrended traces of Per2::luc bioluminescence at various forskolin (FK) concentrations and circadian times (CT) within the shaded region of the phase response curve (PRC) are shown. A red bar represents the timing of FK administration. CT and the amount of phase shift are indicated above the traces. Damped sinusoidal curve fitting before and after FK administration are shown on the right. (PDF) [file pone.0181223.s002.pdf]

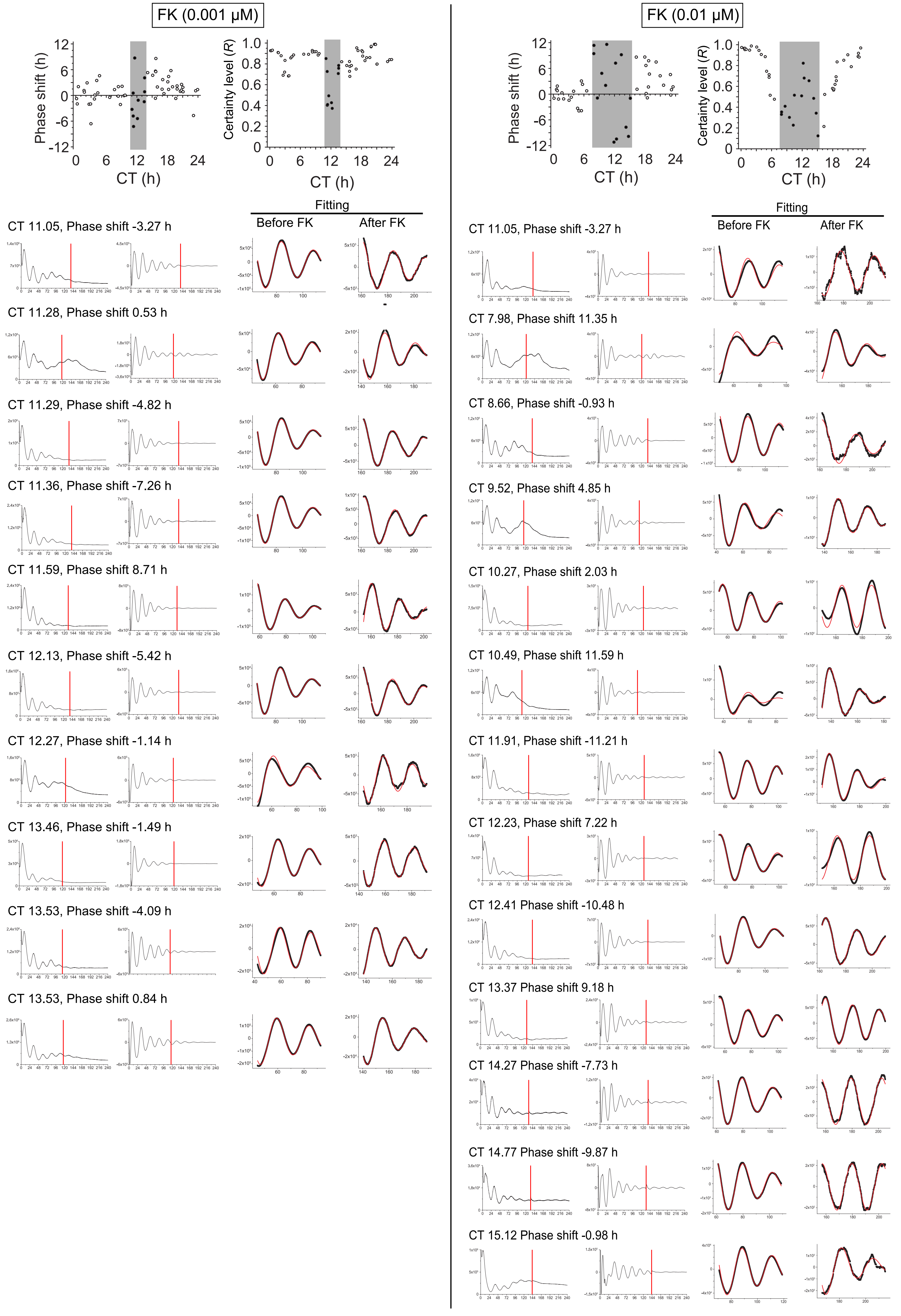

FK (0.001 μM)
Phase shift (h)
CT (h)
Certainty level (R)
Fitting
Before FK
After FK
CT 11.05, Phase shift -3.27 h
CT 11.28, Phase shift 0.53 h
CT 11.29, Phase shift -4.82 h
CT 11.36, Phase shift -7.26 h
CT 11.59, Phase shift 8.71 h
CT 12.13, Phase shift -5.42 h
CT 12.27, Phase shift -1.14 h
CT 13.46, Phase shift -1.49 h
CT 13.53, Phase shift -4.09 h
CT 13.53, Phase shift 0.84 h
FK (0.01 μM)
Phase shift (h)
CT (h)
Certainty level (R)
Fitting
Before FK
After FK
CT 11.05, Phase shift -3.27 h
CT 7.98, Phase shift 11.35 h
CT 8.66, Phase shift -0.93 h
CT 9.52, Phase shift 4.85 h
CT 10.27, Phase shift 2.03 h
CT 10.49, Phase shift 11.59 h
CT 11.91, Phase shift -11.21 h
CT 12.23, Phase shift 7.22 h
CT 12.41 Phase shift -10.48 h
CT 13.37 Phase shift 9.18 h
CT 14.27 Phase shift -7.73 h
CT 14.77 Phase shift -9.87 h
CT 15.12 Phase shift -0.98 h
