# Supplementary material for: Transition of phase response properties and singularity in the circadian limit cycle of cultured cells
Source: PLoS One. 2017 Jul 17;12(7):e0181223. doi: 10.1371/journal.pone.0181223 (PMC5513448; doi:10.1371/journal.pone.0181223)
Supplement: S3 Fig — (PDF) [file pone.0181223.s003.pdf]

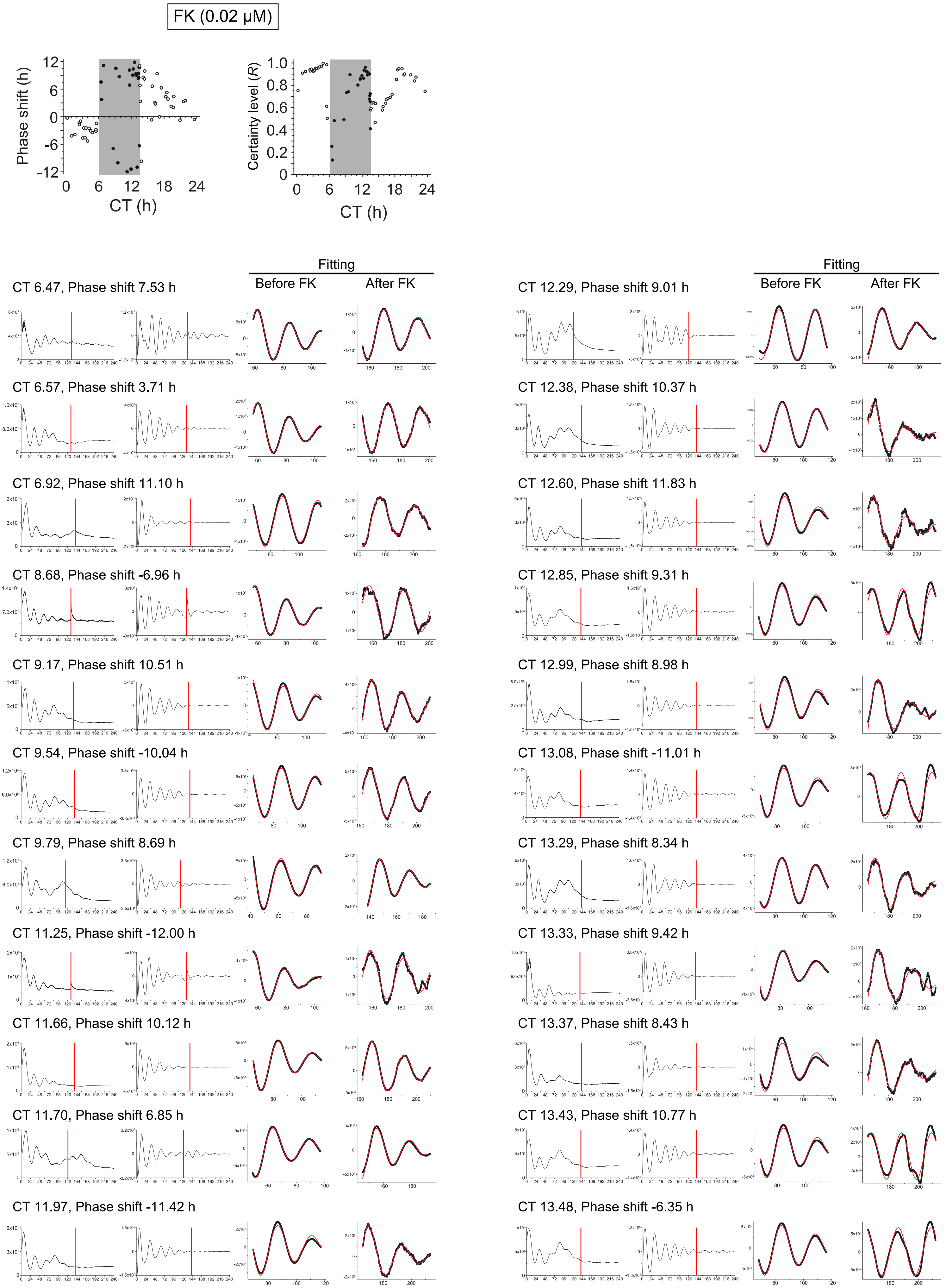

FK (0.02 μM)
Phase shift (h)
CT (h)
Certainty level (R)
CT (h)
Fitting
Before FK
After FK
CT 6.47, Phase shift 7.53 h
CT 6.57, Phase shift 3.71 h
CT 6.92, Phase shift 11.10 h
CT 8.68, Phase shift -6.96 h
CT 9.17, Phase shift 10.51 h
CT 9.54, Phase shift -10.04 h
CT 9.79, Phase shift 8.69 h
CT 11.25, Phase shift -12.00 h
CT 11.66, Phase shift 10.12 h
CT 11.70, Phase shift 6.85 h
CT 11.97, Phase shift -11.42 h
Fitting
Before FK
After FK
CT 12.29, Phase shift 9.01 h
CT 12.38, Phase shift 10.37 h
CT 12.60, Phase shift 11.83 h
CT 12.85, Phase shift 9.31 h
CT 12.99, Phase shift 8.98 h
CT 13.08, Phase shift -11.01 h
CT 13.29, Phase shift 8.34 h
CT 13.33, Phase shift 9.42 h
CT 13.37, Phase shift 8.43 h
CT 13.43, Phase shift 10.77 h
CT 13.48, Phase shift -6.35 h
