# Supplementary material for: Transition of phase response properties and singularity in the circadian limit cycle of cultured cells
Source: PLoS One. 2017 Jul 17;12(7):e0181223. doi: 10.1371/journal.pone.0181223 (PMC5513448; doi:10.1371/journal.pone.0181223)
Supplement: S1 Methods — (PDF) [file pone.0181223.s007.pdf]

S1 Methods. Function fitting to phase response curves. In the experiment, a of phase shifts $\Delta\Phi_i(\Phi_i)$ induced by perturbation applied at phase Φ_i was observed = 1,2,⋯,N). These points were fitted into two types of phase response curve (PRC). Type 1 PRC was characterized as a continuous curve with relatively small phase shifts, whereas type 0 PRC gave rise to a discontinuity at the "breakpoint." By focusing on their differences in continuity, we approximated the observed phase shifts either as type 1 or type 0 PRC.

In type 1 fitting, we simply fitted a cosine function to the phase shift points as

$$\widetilde{\Delta\phi}(\phi_i) = a\sin(2\pi(\phi_i + \alpha)/24) + c, \qquad (1)$$

where the three constants (a,α,c) were determined by minimizing the fitting error $\epsilon = \sum_{i=1}^{N}\left(\Delta\phi_i(\phi_i) - \widetilde{\Delta\phi}(\phi_i)\right)^2$ (Halberg et al., 1967). The fitted PRCs are displayed in Fig 5B.

In type 0 fitting, the phase shift data were transformed as follows: First, the phase shifts induced at phases larger than the breakpoint, denoted as ϕ_b, were shifted by one cycle as

$$\Delta\phi_i^*(\phi_i) = \begin{cases} \Delta\phi_i(\phi_i) & (\phi_i < \phi_b) \\ \Delta\phi_i(\phi_i) - 24 & (\phi_i \geq \phi_b) \end{cases} \qquad (2)$$

We chose the breakpoint $\phi_b \in [0,24]$ in such a way that it provided the minimum

fitting error. Next, a diagonal line (slope of 1) was added to the phase shifts as $\Delta\phi_i^*(\phi_i)+\phi_i$ so that they were distributed on a periodic curve. Then, the periodically distributed points were fitted by a cosine function $a\sin(2\pi(\phi_i+\alpha)/24)+c$. By transforming the fitted cosine function back, the type 0 curve was finally obtained as

$$\widetilde{\Delta\phi}(\phi_i)=\begin{cases} a\sin(2\pi(\phi_i+\alpha)/24)+c-\phi_i & (\phi_i<\phi_b) \\ a\sin(2\pi(\phi_i+\alpha)/24)+c-\phi_i+24 & (\phi_i\geq\phi_b) \end{cases} \qquad (3)$$

The fitted PRCs are displayed in Fig 5A.

To evaluated the fitting accuracy for both type 1 and type 0 PRCs, the normalized root mean square error (*NRMSE*) was calculated for each curve as

$$E_{nrms}=\frac{\sqrt{\sum_{i=1}^{N}\left(\Delta\phi(\phi_i)-\widetilde{\Delta\phi}(\phi_i)\right)^2}}{\sqrt{\sum_{i=1}^{N}\left(\Delta\phi(\phi_i)-\overline{\Delta\phi}\right)^2}} \qquad (4)$$

where $\overline{\Delta\phi}=\frac{1}{N}\sum_{i=1}^{N}\Delta\phi(\phi_i)$.
