# Supplementary material for: Transition of phase response properties and singularity in the circadian limit cycle of cultured cells
Source: PLoS One. 2017 Jul 17;12(7):e0181223. doi: 10.1371/journal.pone.0181223 (PMC5513448; doi:10.1371/journal.pone.0181223)
Supplement: S2 Methods — (PDF) [file pone.0181223.s008.pdf]

S2 Methods. Minimum embedding dimension of experimental time series data. In this analysis, we supposed that the signal $\{x(t): t = 1, \dots, N\}$ was generated from deterministic nonlinear dynamics forming a low-dimensional attractor. According to Takens [1] and Sauer *et al*. [2], an attractor, which is topologically equivalent to the original, can be reconstructed in the following delay-coordinate space if the embedding dimension d is high enough:

$$\mathrm{X}(t) = (x(t), x(t-\tau), \dots, x(t-(d-1)\tau)), \qquad (1)$$

The time lag τ can be chosen arbitrarily. In order to determine the minimum embedding dimension d_E, which provides a number of degrees of freedom for the underlying dynamics, the false nearest neighbor (FNN) method [3] was utilized. The FNN algorithm focused on a topological change in the reconstructed dynamics in the delay-coordinate space as follows. Consider a trajectory $\{X(t)\}$ reconstructed in the delay-coordinate space of Eq. (1) with an embedding dimension d. Each data point, $\mathrm{X}(t)$, denoted its rth nearest neighbor by $\mathrm{X}(t_r)$. The square of the Euclidean distance between $\mathrm{X}(t)$ and $\mathrm{X}(t_r)$ was then given by

$$R_d^2(t, r) = \|\mathrm{X}(t) - \mathrm{X}(t_r)\|^2 = \sum_{k=0}^{d-1}[x(t - k\tau) - x(t_r - k\tau)]^2. \qquad (2)$$

Let us see a change in the distance R_d when the embedding dimension was increased as $d \to d + 1$. Addition of the new $(d + 1)$-th coordinate increased the distance between $X(t)$ and $X(t_r)$ by

$$R_{d+1}^2(t,r) = R_d^2(t,r) + [x(t-d\tau) - x(t_r - d\tau)]^2, \qquad (3)$$

If the increase in the distance from $R_d(t)$ to $R_{d+1}(t)$ was significantly large as

$$\left[\frac{R_{d+1}^2(t,r) - R_d^2(t,r)}{R_d^2(t,r)}\right]^{1/2} > R_{\mathrm{tol}} \quad (R_{\mathrm{tol}}\text{ : threshold value}), \quad (4)$$

then $X(t_r)$ can be considered as a "false" nearest neighbor to $X(t)$ caused possibly by a self-crossing of the orbit in the d-dimensional delay-coordinate space. Hence condition (4) provides a first criterion for FNNs.

There was a second criterion for FNNs. Because we dealt with finite data points, the trajectory distribution can be sparse in the delay-coordinate space and some nearest neighbors to $X(t)$ might not be so close, i.e., $R_d(t) \approx R_A$ (R_A: an attractor size). If such distant nearest neighbors are FNNs, the addition of a new (d + 1)-th coordinate may stretch their distances by the attractor size and will result in $R_{d+1}(t) \approx 2R_A$. Hence, for such distant neighbors, the second criterion for false neighbors was given by

$$\frac{R_{d+1}(t)}{R_A} > 2, \qquad (5)$$

where the attractor size R_A can be computed as

$$R_A^2 = \frac{1}{N_{\mathrm{data}} - (d-1)\tau} \sum_{t=1+(d-1)\tau}^{N_{\mathrm{data}}} \| x(t) - \bar{x} \|^2, \qquad (6)$$

$$\bar{x} = \frac{1}{N_{\mathrm{data}} - (d-1)\tau} \sum_{t=1+(d-1)\tau}^{N_{\mathrm{data}}} x(t). \qquad (7)$$

The FNN was finally defined as the nearest neighbor that satisfied either of the first criterion (4) or the second criterion (5).

References

1. Takens F. Detecting strange attractors in turbulence. In: Rand DA, Young LS, editors. Dynamical Systems and Turbulence, Lecture Notes in Mathematics. 898. Berlin: Springer; 1981. p. 366-81.

2. Sauer T, Yorke JA, Casdagli M. Embedology. J Stat Phys. 1991;65(3-4):579-616. doi: Doi 10.1007/Bf01053745. PubMed PMID: ISI:A1991GR56900009.

3. Kennel MB, Brown R, Abarbanel HD. Determining embedding dimension for phase-space reconstruction using a geometrical construction. Physical review A. 1992;45: 3403-11. PubMed PMID: 9907388
